# Enhancing sugar beet growth and quality using green-synthesized nanofertilizers: a comparative study of seed priming and foliar spraying

**DOI:** 10.1186/s12870-025-07308-w

**Published:** 2025-09-23

**Authors:** Marwa M.A. Ghallab, Ahmed M. Abdelfatah, Manal Fawzy

**Affiliations:** 1 Breeding and Genetic Department, Agricultural Research Center, Sugar Crops Research Institute, Giza, Egypt; 2 Green Technology Group, Environmental Sciences Department, Faculty of Science, Alexandria University, Alexandria, 21511 Egypt

**Keywords:** Green-synthesized nanofertilizers, Silver nanofirtilizers, Zinc oxide nanofertilizers, Sugar beet cultivars., Seed priming, Foliar application

## Abstract

This study evaluated the effects of foliar spraying and seed priming with green-synthesized nanofertilizers (NFs) containing silver (Ag), zinc oxide (ZnO), and an Ag/ZnO composite on the growth and quality of two sugar beet (*Beta vulgaris L*.) varieties, Kawemira and Dema Poly. The experiment was conducted under greenhouse conditions at El-Sabahia Research Station, Alexandria, Egypt (31°12′N, 29°58′E) using a randomized complete block design (RCBD) with five replicates in 50 cm earthenware pots. Treatments included foliar sprays or seed priming at 0 (control), 50, and 100 ppm, applied at different growth stages. Foliar application of AgNFs at 100 ppm, 15 days after sowing, achieved the highest fresh shoot weights (240 g in Kawemira, 231 g in Dema Poly) and root weights (995.6 g in Kawemira, 984 g in Dema Poly). Seed priming with Ag/ZnONFs composite at 50 ppm for 24 h resulted in high shoot weights (240 g and 241 g) and root weights (902 g and 865 g) for Kawemira and Dema Poly, respectively. Foliar spraying with Ag/ZnONFs composite at 50 ppm increased total soluble solids (TSS) to 28.8% (Kawemira) and 30.8% (Dema Poly), and sucrose content to 21.25% and 22.25%, respectively. The formation and elemental composition of AgNFs, ZnONFs, and Ag/ZnONFs composites were confirmed via scanning electron microscopy (SEM) and energy-dispersive X-ray spectroscopy (EDS). These findings demonstrate that targeted use of green-synthesized nanofertilizers can significantly improve sugar beet growth, sugar content, and juice quality. The results highlight the potential of nanofertilizer application particularly seed priming as a sustainable and cost-effective approach for enhancing crop productivity.

## Introduction

Food security has become a significant global concern as the world’s population grows, and the present climate change catastrophe worsens. The use of conventional agrochemicals to boost agricultural production has degraded fertile soil, polluted the environment, and posed health concerns to humans and agroecosystems [1–4]. Nanotechnology in agriculture is a rapidly expanding and novel area of research that aims to improve crop yield and nutrient use efficiency by using nano-sized agrochemicals at lower doses than conventional agrochemicals. In agriculture, nanoparticles (NPs) are used as both fertilizers and insecticides. Nano-based agricultural supplements have been found to have positive effects on plant development [5].

Agri-nanotechnology, an integration of nanoscience with agriculture has emerged as a transformative approach for promoting sustainable farming and enhancing global food security [6]. It involves the application of nanomaterials to improve crop productivity, soil health, and resource efficiency. Key applications include nanofertilizers for targeted nutrient delivery and nanosensors for real-time monitoring of plant health, soil conditions, and environmental factors [4, 7]. These technologies enable precision farming, helping to optimize inputs, conserve resources, and minimize environmental impacts. Additionally, nanomaterials such as nanoparticles and nanocomposites play a significant role in crop protection due to their unique optical properties, high surface area, and size-dependent behavior [8, 9]. As interdisciplinary research progresses, nanotechnology continues to offer promising solutions in plant nutrition and defense.

Nanotechnology has become increasingly integrated into agriculture, offering innovative solutions to enhance crop productivity and sustainability [10]. In particular, the use of nanofertilizers has shown significant promise in improving the growth, yield, and quality of sugar beet crops by enhancing nutrient efficiency and plant development [11, 12]. Studies have demonstrated that micronutrient-based fertilization, including nanoscale forms, can stimulate root and sugar production in sugar beet plants. Silver nanoparticles (AgNPs), in particular, have been found to enhance photosynthetic efficiency and resource utilization, especially under saline conditions, thereby increasing plant resilience and competitiveness [13]. Moreover, the application of AgNPs to *Satureja hortensis* notably improved seed germination and overall plant growth in greenhouse trials, creating more favorable conditions for early plant development [14].

Among various nanomaterials, zinc oxide (ZnO) and silver nanoparticles (AgNPs) have garnered significant attention for their potential as eco-friendly and efficient nanofertilizers. These nanoparticles enhance nutrient uptake, promote seed germination, and improve physiological traits such as sugar content and pigment levels in crops [15]. Their effectiveness, however, depends on factors such as particle size, composition, plant species, and application concentration. ZnONPs, in particular, have shown promising results in mitigating yield loss due to climate stress, making them a cost-effective and sustainable tool for crop enhancement [16, 17]. AgNPs also play a critical role by enhancing photosynthesis, improving stress tolerance, and supporting early plant development. The shift toward green and sustainable nanofertilizers like ZnONPs and AgNPs aligns with the principles of smart and organic agriculture, minimizing environmental risks associated with conventional agrochemicals [18, 19]. The function of NPs as secondary metabolite elicitors is highly dependent on their chemical compositions and physical properties, as well as the concentration of application; thus, extensive dose-response studies are required to determine the optimal concentration for each NP-plant system to maximize secondary metabolite yield while minimizing toxic effects on the plant, consumers, and the environment [20].

Agricultural development is vital to economic growth and food security, especially amid growing environmental challenges. Nanotechnology offers a promising solution by improving resource efficiency, boosting productivity, and minimizing environmental impact in the agri-food sector. However, conventional methods of nanoparticle synthesis may harm ecosystems. As a sustainable alternative, green synthesis using plant-based protocols has emerged, producing biocompatible nanoparticles. Among the key applications, nanofertilizers stand out for their high surface area and ability to deliver nutrients efficiently. Additionally, nanoparticles serve as targeted carriers for agrochemicals, enhancing nutrient delivery and crop protection [5].

Sugar beet (*Beta vulgaris L.*) is a large root crop grown all over the world and used for sugar extraction, producing millions of tons of sugar for human consumption and beet pulp for animal feed per year [21]. Its significance arises from its adaptability to varied settings, making it a desirable commercial crop, especially in newly reclaimed regions, and its high sugar yield potential [22].

Based on the premise that nanofertilizers can provide controlled nutrient release, improve nutrient uptake efficiency, and minimize environmental losses, we hypothesized that green-synthesized zinc oxide (ZnO), silver (Ag), and composite ZnO/Ag nanofertilizers (NFs), applied through seed priming or foliar spraying at varying concentrations, would differentially enhance growth, yield components, and juice quality of sugar beet varieties under controlled conditions. Accordingly, the primary objective of this study is to explore the transformative potential of these green-synthesized nanofertilizers in advancing agricultural productivity and sustainability. Specifically, this research evaluates the comparative effects of the two application methods on growth and quality parameters of two sugar beet varieties, aiming to address critical challenges in modern agriculture, including nutrient loss, environmental impact, and crop yield optimization, while contributing to the development of sustainable practices with reduced ecological footprints.

## Materials and methods

### Materials

All chemicals and reagents utilized in this study were of analytical grade. Deionized water with a resistivity of 18.2 MΩ/cm, obtained from a Milli-Q system (Arioso Power II, Korea), was employed for the preparation of all aqueous solutions used throughout the experimental procedures. Zinc nitrate [Zn(NO_3_)_2_.6H_2_O, >99.5%)], Silver nitrate [AgNO_3_, >99.5%)], Sodium hydroxide (NaOH, 97%), ethanol (C_2_H_5_OH, 99.9%), and hydrochloric acid (HCl, 37%) were purchased from Merck, US.

### Preparation of plant extract

Leaves of *Beta vulgaris* (sugar beet) were washed several times using tap water and distilled water to remove any impurities. Subsequently, plant leaves were shredded into small pieces and dried in a laboratory oven at 60 °C for 2 h until constant weight was reached. Afterwards, 5 g of plant fine powder was soaked in 100 mL distilled water and gently stirred at 70 ℃ for 20 min, producing a reddish-brown extract. The extract was then filtered and stored in sterilized falcon tubes in a refrigerator at 4 ℃ for subsequent use.

### Green synthesis of ag, zno, and ag/zno nanofertilizers

**Fig. 1:**
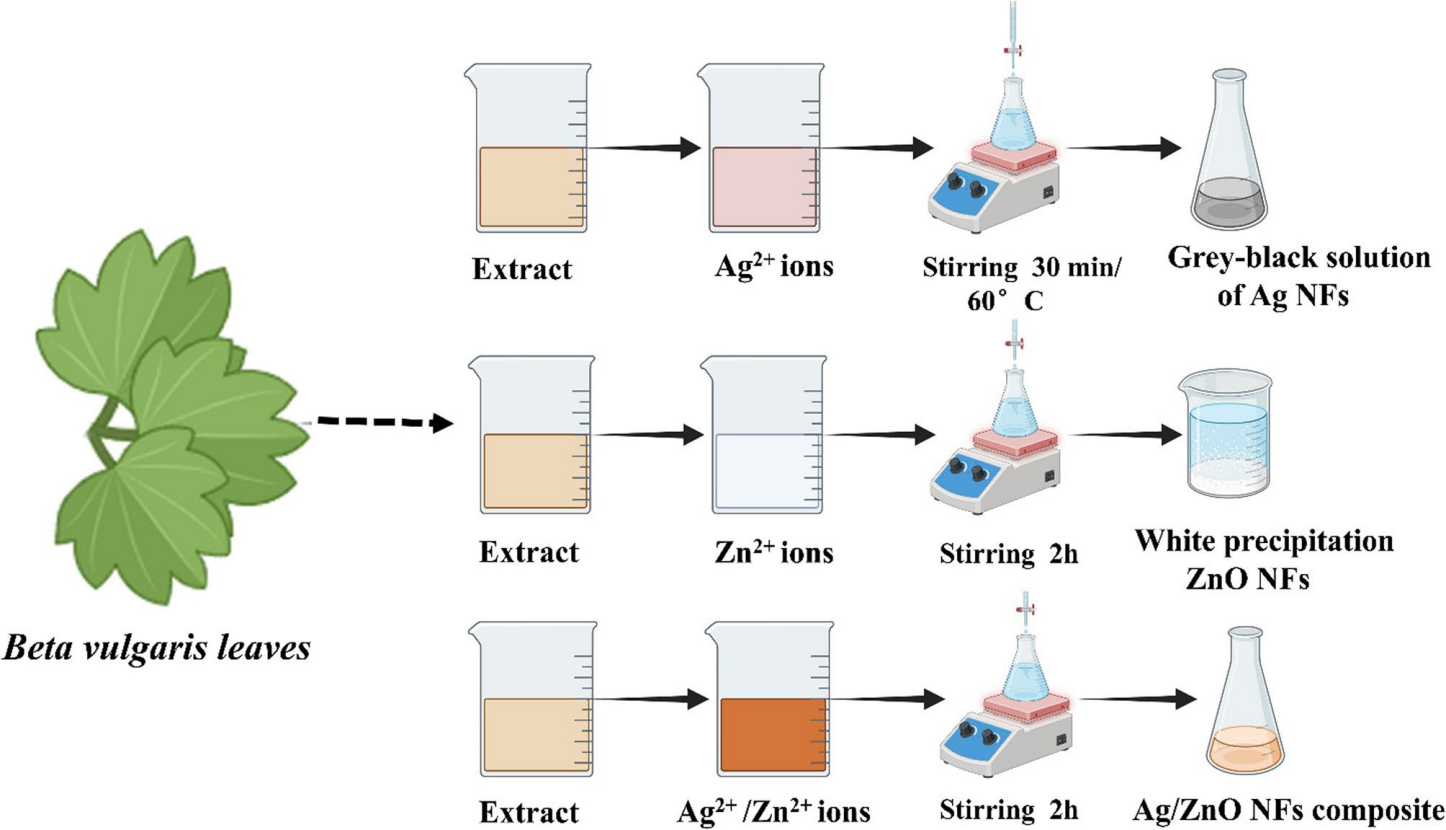
Schematic representation for the green synthesis of AgNFs, ZnONFs, and Ag/ZnONFs composite

The green synthesis of silver nanofertilizers (AgNFs) was conducted following the method described by Garibo, Borbón-Nuñez [23] with slight modifications. To prepare solutions containing 50 ppm and 100 ppm of Ag nanofertilizers, 0.018 g and 0.037 g of silver nitrate, respectively, were dissolved in 250 mL of distilled water, with the pH adjusted to 4.5 ± 0.1. Subsequently, 25 mL of plant leaf extract was added drop by drop to the silver ion solutions under vigorous stirring at 60 °C for 30 min. The solution exhibited a grey-black color, indicating the formation of AgNFs (Fig. 1).

The synthesis of zinc oxide nanofertilizers (ZnONFs) was conducted according to the procedure outlined by Naiel, Fawzy [24]. For the preparation of solutions containing 50 ppm and 100 ppm of ZnONFs, 0.11 g and 0.055 g of zinc nitrate, respectively, were dissolved in 50 mL of distilled water. Subsequently, 25 ml of plant leaf extract was added to the solutions under vigorous stirring for 2 h. A white precipitate was formed, indicating the successful formation of ZnONFs.

The nanofertilizers composite of Ag/ZnO was synthesized by combining equal amounts of silver nitrate and zinc nitrate, as described previously, in 250 mL of distilled water. The mixture was stirred together for 2 h. The plant extract was then added drop by drop at room temperature until a color change was observed, indicating the formation of the Ag/ZnONFs composite as represented in Fig. 1.

### Characterization of green synthesized NFs

The green synthesized nanofertilizers (NFs) were characterized by Scanning Electron Microscopy (SEM-S4800, Hitachi), and Energy Dispersive X-ray Spectroscopy (EDX-JEOL model JSM-IT100).

### Experimental study

The experiment was conducted in a greenhouse at El-Sabahia Research Station (latitude of 31^ᵒ^12΄N and longitude of 29^ᵒ^58΄E), Alexandria, Agriculture Research Center, Egypt. The seeds of the commonly cultivated, in Egypt, sugar beet varieties namely: Kawemira and Dema poly were used. Both varieties were obtained from the Sugar Crops Research Institute (SCRI) at the Agricultural Research Center (ARC) in Giza, Egypt. However, the origin of Dema poly is France, while Kawemira is a German variety. Two sugar beet varieties, Kawemira and Dema poly, were employed. Two different approaches were adopted: foliar spraying and seed priming with green synthesized AgNFs, ZnONFs, and Ag/ZnONFs composite. These treatments were administered at three different concentrations: 0 ppm (control), 50 ppm, and 100 ppm.

Foliar spray of AgNFs at concentrations of 50 ppm (S1) and 100 ppm (S2), foliar spray of zinc oxide NFs at concentrations of 50 ppm (S3) and 100 ppm (S4), and foliar spray of Ag/ZnONFs composite at concentrations of 50 ppm (S5) and 100 ppm (S6), a control treatment involving water foliar spray (C). Each treatment was applied to the plants twice on three different dates: 15, 30, and 60 days after sowing as represented in Table 1.

**Table 1:** Description of the experimental treatments applied to two sugar beet varieties, including types of nanofertilizers, concentrations, application methods (foliar spray or seed priming), and application timings

Application methods	varieties	Timing	Treatment	Concentration(ppm)	Code
Foliar Spray	Kawemira and Dema Poly	15,30, and 60 days after swing	Water	-	C
			AgNFs	50	S1
			AgNFs	100	S2
			ZnONFs	50	S3
			ZnONFs	100	S4
			Ag/ZnONF	50	S5
			Ag/ZnONF	100	S6
Seed Priming	Kawemira and Dema Poly	6,12 and 24 h before sowing	water	-	C
			AgNFs	50	T1
			AgNFs	100	T2
			ZnONFs	50	T3
			ZnONFs	100	T4
			Ag/ZnONFs	50	T5
			Ag/ZnONFs	100	T6

#### Seed priming

Likewise, different concentrations of Ag, ZnONFs, and Ag/ZnONFs composite were applied. Pre-sowing seed treatments C = Priming with tap water, T1 = Priming with AgNFs (50 ppm), T2 = Priming with AgNFs (100 ppm), T3 = Priming with ZnONFs (50 ppm), T4 = Priming with ZnONFs (100 ppm), T5 = Priming with Ag/ZnONFs composite (50 ppm) & T6 = Priming with Ag/ZnONFs (100 ppm). Seed priming was conducted by immersing the required quantity of sugar beet seeds in tap water and nanofertilizer solutions at various concentrations for different durations (6, 12, and 24 h) for each treatment, using a ratio of 1:1 (grams of seeds to volume of solution). Subsequently, the treated seeds were air-dried in the shade to maintain optimal seed moisture levels.

In both treatments, a randomized complete block design (RCBD) with five replicates was employed. Sugar beet seeds of the selected varieties were planted in No. 50 earthenware pots on 5 November 2023. Each pot contained 30 kg of soil sourced from Sabahia Research Station, Alexandria, Egypt. The experiment was conducted in a greenhouse maintained at 24 ± 2 °C during the day and 18 ± 2 °C at night, with a relative humidity of 60 ± 5% and a photosynthetic photon flux density (PPFD) of approximately 350 µmol m⁻² s⁻¹ at canopy height. The physical and chemical properties of the experimental soil and irrigation water are presented in Table 2. A basal dressing consisting of 4.8 g of urea (46.5%), equivalent to approximately 179 kg of nitrogen per hectare, along with 10.5 g of potassium dihydrogen phosphate and 9 g of calcium superphosphate (15.5%), representing about 114 kg each of potassium oxide and phosphorus pentoxide per hectare, respectively, was applied.

**Table 2:** Physical and chemical properties of the experimental soil and irrigation water

Parameter	Soil	Irrigation water
Texture	Sandy	-
**pH**	8.32	7.23
**EC (dS m⁻¹)**	2.12	0.52
**Soluble cations (meq L⁻¹)**		
Ca²⁺	6.70	—
Mg²⁺	2.72	—
Na⁺	9.71	—
K⁺	2.11	—
**Soluble anions (meq L⁻¹)**		
HCO₃⁻	3.77	—
SO₄²⁻	6.93	—
Cl⁻	10.64	—

### Data recording

The relative chlorophyll contents were performed 90 days after sowing. Relative chlorophyll contents were measured using a portable chlorophyll meter (Minolta SPAD-502, Japan), at three points (upper, middle, and lower parts) of each leaf, and averaged to represent the individual measurement of a leaf.

Harvesting was conducted on June 5th, marking the culmination of the growing season 210 days after sowing. Plants from each treatment were collected to assess total biomass, fresh shoot, and root weight. Following leaf removal, the roots were meticulously washed, and various plant parts were individually weighed. Quality parameters such as total soluble solids (TSS) percentage were determined in fresh roots using a hand refractometer, following the method outlined by Cooke and Scott [25]. Sucrose content: Determined using a saccharometer on a lead acetate extract obtained from freshly macerated roots, following the method described by Carruthers and Oldfield [26]. Purity%: Calculated according to Eq. 1 proposed by Carruthers, Oldfield [27]:

1 $$Purity\%\;=\;(Sucrose/TSS)\times100$$

### Statistical analysis

Statistical analyses were conducted on the growth and juice quality traits using the methods outlined by Snedecor and Cochran [28]. The analysis of variance (ANOVA) technique was applied to evaluate the effects of treatments on the measured parameters. Mean comparisons were conducted using the Revised Least Significant Difference (L.S.D.) test at the 5% significance level according to Waller and Duncan [29]. Pearson correlation analysis was also performed to examine the relationships among the measured variables. All statistical analyses were carried out using CoStat software (version 6.45) and Microsoft Excel.

## Results and discussion

### Characterization results

#### Surface morphology of nanofertilizers

**Fig. 2:**
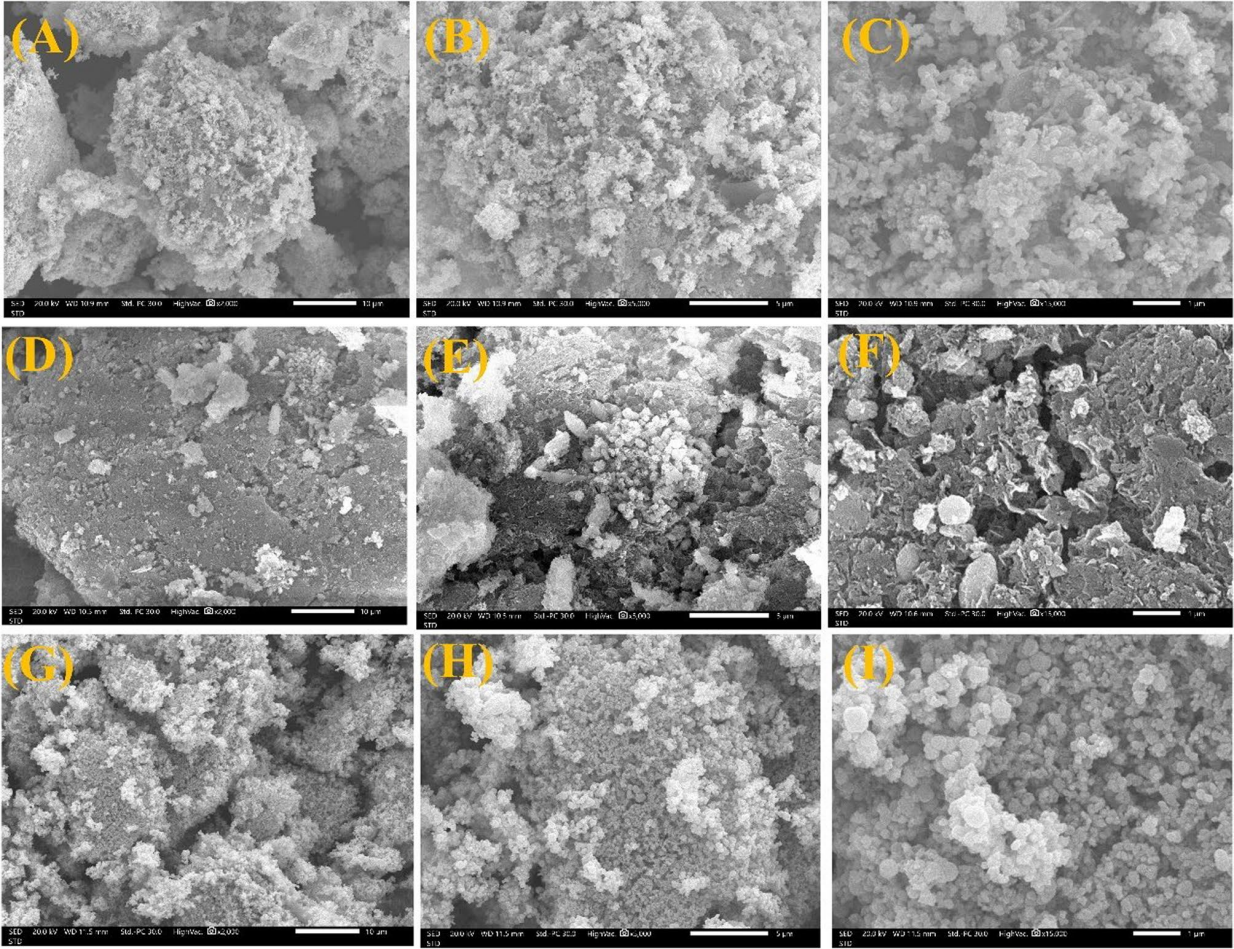
SEM images for AgNFs 50 ppm (A), and 100 ppm (B, C), ZnONFs 50 ppm (D), and 100 ppm (E, F), and Ag/ZnONFs composite 50 ppm (G), and 100 ppm (H, I)

Sanning electron microscopy (SEM) analysis revealed distinct morphological features in green-synthesized Ag, ZnO, and Ag/ZnO composite NFs at concentrations of 50 ppm and 100 ppm (Fig. 2). At 50 ppm, AgNFs formed loosely aggregated, cauliflower-like structures composed of nanoscale particles (30–100 nm) with a porous, sponge-like texture (Fig. 2A). This unique morphology provides enhanced surface roughness and high surface area, which may improve nanoparticle adhesion to plant surfaces and facilitate controlled silver ion release during foliar application. The observed partial aggregation is characteristic of silver nanoparticles due to their high surface energy. In contrast, the 100 ppm AgNFs exhibited a more densely packed arrangement of ultra-fine spherical and semi-spherical nanoparticles (20–80 nm), demonstrating improved nucleation and particle uniformity at higher concentrations as shown in Fig. 2 (B, C). This compact, homogeneous nanostructure with minimal voids suggests potentially enhanced bioavailability, reactivity, and antimicrobial efficacy for agricultural applications. The 50 ppm ZnONFs appeared as well-dispersed spherical nanoparticles (20–50 nm) with only mild agglomeration (100–300 nm clusters) (Fig. 2D). Their smooth surfaces indicate stable synthesis conditions favorable for controlled nutrient release. At 100 ppm, while the primary particles maintained their spherical morphology, significantly larger aggregates (200–500 nm) formed, emphasizing the need for dispersion-enhancing strategies at elevated concentrations as represented in Fig. 2 (E, F). The composite material at 50 ppm showed Ag nanoparticles (10–30 nm) evenly distributed on ZnO spheres (20–50 nm), with moderate clustering (100–300 nm) (Fig. 2G). This hybrid structure effectively combines the beneficial properties of both components. Even at 100 ppm, the composite maintained its structural integrity, forming denser Ag-ZnO clusters (200–500 nm) while preserving the interfacial stability between components, demonstrating robust composite formation across concentration ranges [Fig. 2 (H, K)].

#### Energy-dispersive X-ray spectroscopy (EDS)

**Fig. 3:**
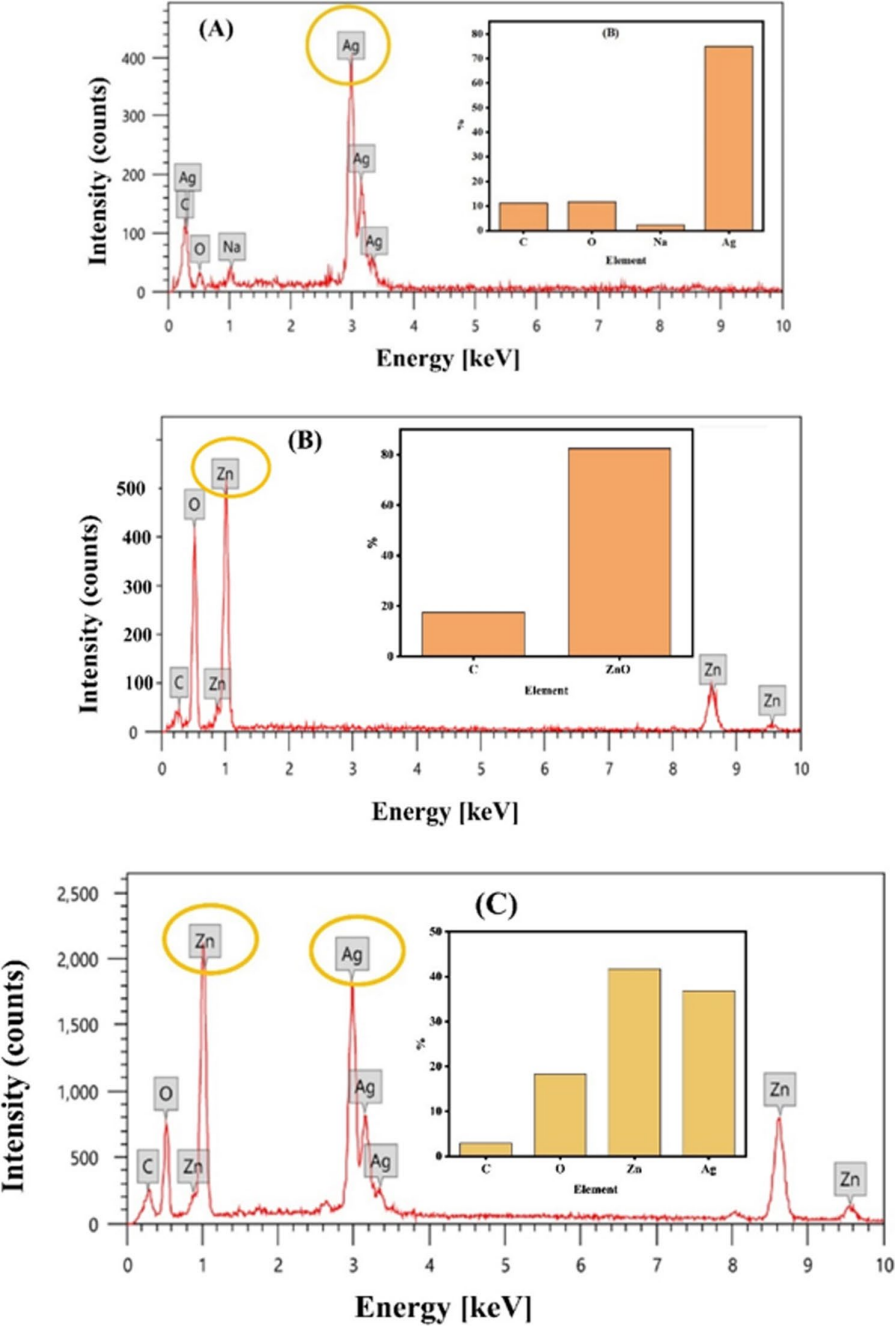
EDX analysis for AgNFs **A** ZnONFs **B** Ag/ZnONFs composite **C**. Inset represents the distribution percentage of elements in the sample

EDS confirmed the elemental composition and purity of the green synthesized nanofertilizers (Ag, ZnO, and Ag/ZnO composite). For the AgNFs, the EDS spectrum (Fig. 3A) revealed a predominant Ag peak (74.87%), confirming successful NFs formation. The detected carbon (11.13%), oxygen (11.73%), and sodium (2.26%) signatures originate from the organic constituents of the plant extract used in the green synthesis process [30]. Notably, the absence of peaks at 3.35 keV (characteristic of Ag₂O) confirms the metallic nature of the synthesized AgNFs, with no detectable oxide formation or other impurities. The ZnONFs exhibited strong zinc (82.45%) and oxygen signals in their EDS spectrum (Fig. 3B), with a Zn: O atomic ratio of approximately 1:1, consistent with stoichiometric ZnO formation [31]. The residual carbon signal (17.55%) is attributed to organic residues from the plant-mediated synthesis. The Ag/ZnO composite spectrum (Fig. 3C) demonstrated the expected elemental composition with Zn (43.3%), Ag (34.61%), and O (18.17%) as primary components. The measured atomic percentages confirm the successful formation of a bimetallic composite, while the minor carbon content (3.93%) reflects residual organic matter from the synthesis process. The absence of unexpected elemental peaks in all samples indicates high purity of the synthesized nanomaterials.

### Effect of nanofertilizers on the growth of sugar beets

**Fig. 4:**
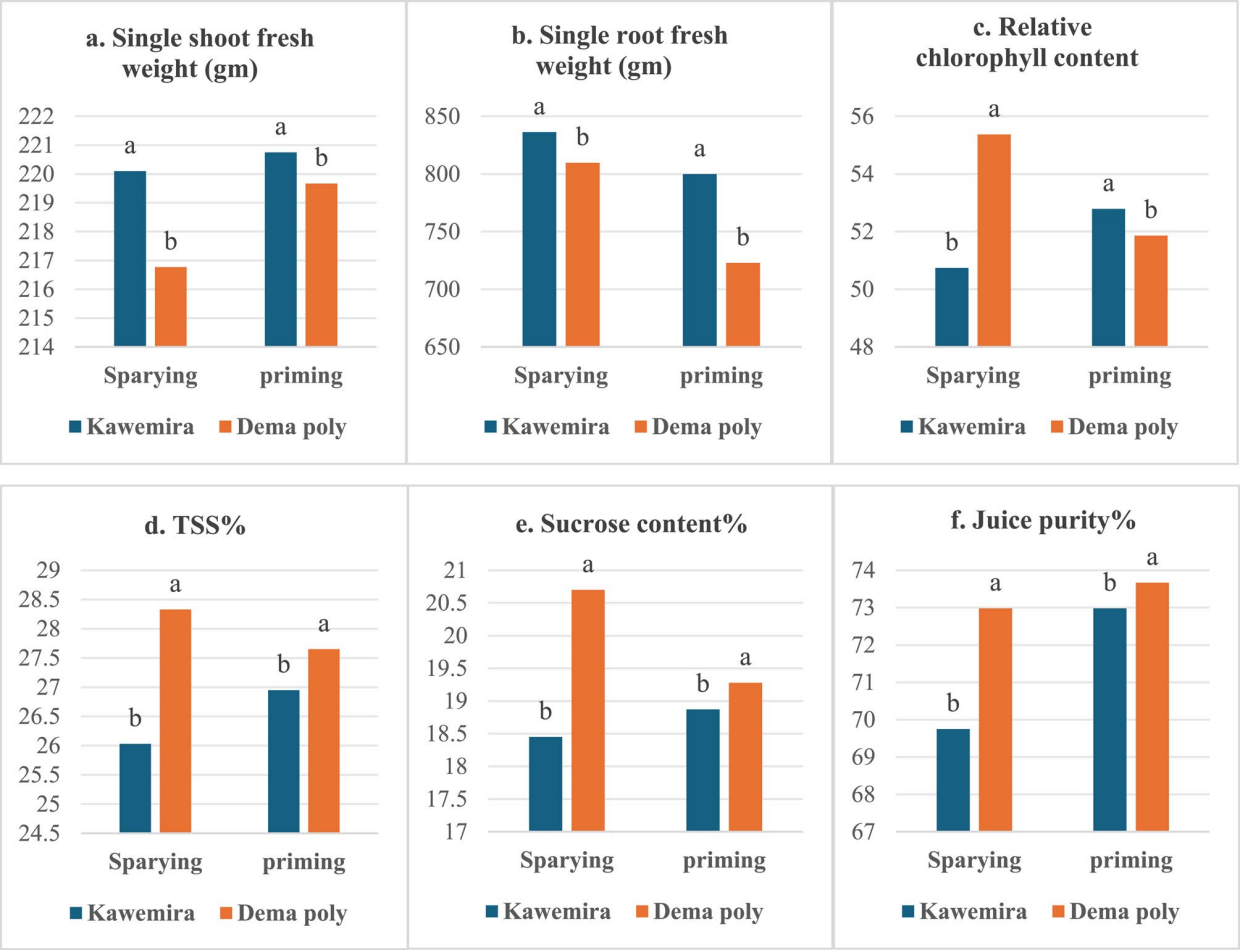
Effect of sugar beet varieties and type of nanofertilizers and treatments, by foliar spraying or seed priming, on single fresh weight of shoot and root, relative chlorophyll content, TSS, sucrose content, and juice purity

Data presented in Fig. 4 indicated that the differences between sugar beet varieties were significant for all studied traits when using green-synthesized nanofertilizers through either foliar spraying or seed priming. The variety Kawemira exhibited the highest weight of a single fresh shoot and root (836.24, 799.89 g) in spraying and priming applications, respectively, while the variety Dema poly showed the highest percentages of total soluble solids (TSS), sucrose content, and juice purity in both spraying and priming applications (Fig. 4). Variation in gene structure among sugar beet varieties contributed to differences in yield and quality, as reported by Alla, Mohamed [32]. Similar findings were observed by Erciyes, Bulut [33], and Abd El-Lateef, Shalabi [34].

The standard deviation was represented for each column. Means with the same letters are not significant according to Duncan’s test.

Figure 4. Effect of sugar beet varieties and type of nanofertilizers and treatments, by foliar spraying or seed priming, on single fresh weight of shoot and root, relative chlorophyll content, TSS, sucrose content, and juice purity.

The analysis of variance (ANOVA) results for the two sugar beet varieties are presented in Table 3. Foliar spraying or seed priming with green-synthesized Ag, ZnONFs, and their Ag/ZnO composite significantly affected most studied traits. Specifically, treatment (T), application date (D), treatment-by-date interaction (T×D), and variety-by-treatment interaction (V×T) showed significant effects on all traits except juice purity (%) under foliar spraying (Table 3). For foliar spraying, variety (V) and the three-way interaction (V×T×D) were also significant. In seed priming (Table 4), treatment (T), date (D), variety (V), and the treatment-by-date interaction (T×D) all significantly influenced the measured traits.

**Table 3:** Analysis of variance of foliar spraying by nanofertilizers ag, znonfs, and ag/znonfs composite on the investigated parameters of the studied sugar beet varieties at three dates

Sources of changes	Mean of squares (MS)					
	Shoot fresh weight/gm	Root fresh weight/gm	Relative chlorophyll content	TSS%	Sucrose content%	Juice purity%
Blocks	50.34 ns	214.10 ns	35.36**	3.61 ns	1.29 ns	33.66 ns
Dates(D)	134.86 *	24127.47**	0.98*	20.09**	11.61**	34.00 ns
Treatments of NFs(T)	1477.96**	140966.16**	154.09**	57.86**	36.63**	23.92ns
Varieties(V)	583.33**	37120.30**	1127.06**	278.88**	280.72**	634.06**
T*D	75.25**	10736.83**	53.44**	12.89**	8.89**	42.79 ns
V*D	6.17 ns	2610.03**	40.58**	3.49 ns	7.08**	75.36 ns
V*T	101.9**	4997.73**	83.63**	4.97**	4.51**	28.79ns
V*T*D	44.97**	3547.18**	24.51**	3.74**	3.72**	85.50*

**,* significant difference at the probability level of 5% and 1% respectively.

**Table 4:** Analysis of variance of seeds priming with nanofertilizers ag, znonfs, and ag/znonfs composite on the investigated parameters of the studied sugar beet varieties at three dates

Sources of changes	Mean of squares (MS)					
	Shoot fresh weight/gm	Root fresh weight/gm	Relative chlorophyll content	TSS%	Sucrose content%	Juice purity%
Blocks	67.86***	52.69 ns	38.96**	6.83**	0.01 ns	0.0004ns
Dates(D)	2049.43***	138456.03**	405.96**	127.72**	73.28**	0.0009*
Treatments of NFs(T)	1391.05**	48631.74**	131.15**	45.86**	18.78**	0.0066**
Varieties(V)	61.88***	311580.58**	45.82**	26.14**	8.85**	0.0025**
T*D	127.35***	5641.43**	40.74**	14.74**	7.84**	0.0025**
V*D	146.67***	18853.03**	1.53 ns	2.79**	0.93*	0.0005ns
V*T	123.1635***	4086.1540**	2.1178*	0.3156ns	0.1313ns	0.0007**
V*T*D	31.8658***	1383.6611**	3.2871**	0.8425*	0.3852ns	0.0005**

**,*significant difference at the probability level of 5% and 1% respectively.

**Fig. 5:**
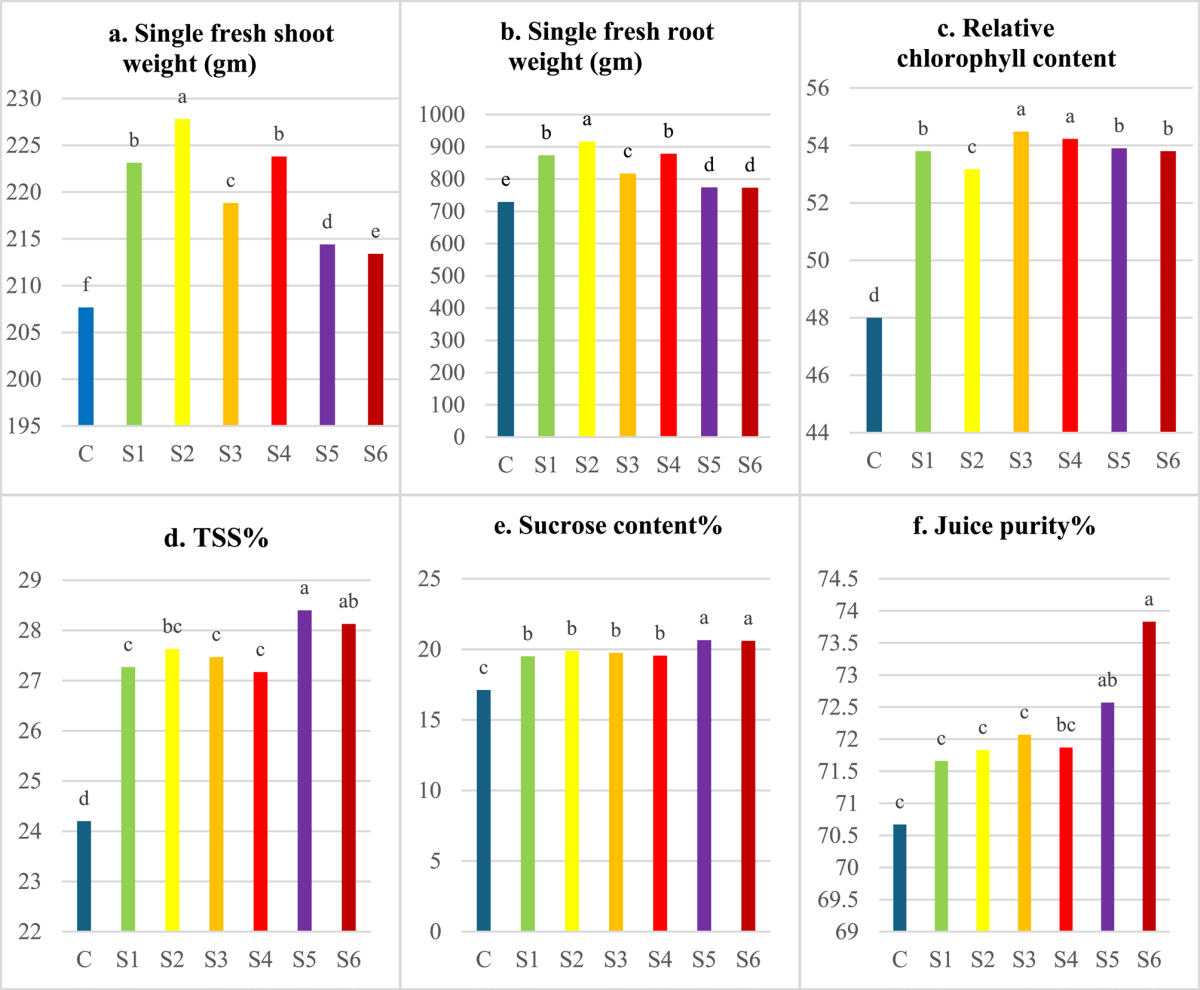
Effect of foliar application of Ag, ZnONFS and Ag/ZnONFs composite on single fresh weight of root and shoot, Chlorophyll content, TSS %, sucrose %, and purity % in two sugars beets genotypes. The vertical bar represents ± SE of means. Water foliar spray (C), foliar spray of AgNFS of 50 ppm (S1) and 100 ppm (S2), foliar spray ZnONFS of 50 ppm (S3) and 100 ppm (S4), foliar spray (Ag/ZnONFs composite) of 50 ppm (S5) and 100 ppm (S6)

The data presented in Fig. 5 shows that foliar application of green-synthesized nanofertilizers (Ag NFs, ZnONFs, and Ag/ZnONFs composite) significantly affected all measured traits of sugar beet at the 5% level (*p* ≤ 0.05), with the control group consistently showing the lowest values. Among foliar treatments, S2 (AgNFs at 100 ppm) resulted in the highest fresh weights of shoots and roots, followed by S1 (AgNFs at 50 ppm). Compared to the control, the 100 ppm Ag NFs treatment increased root and shoot fresh weight by 25.76% and 9.69%, respectively. Relative chlorophyll content increased significantly with AgNFs at 50 ppm, outperforming the 100 ppm treatment. Additionally, ZnONFs treatments (S3: 50 ppm and S4: 100 ppm) showed elevated chlorophyll levels compared to other treatments.

**Fig. 6:**
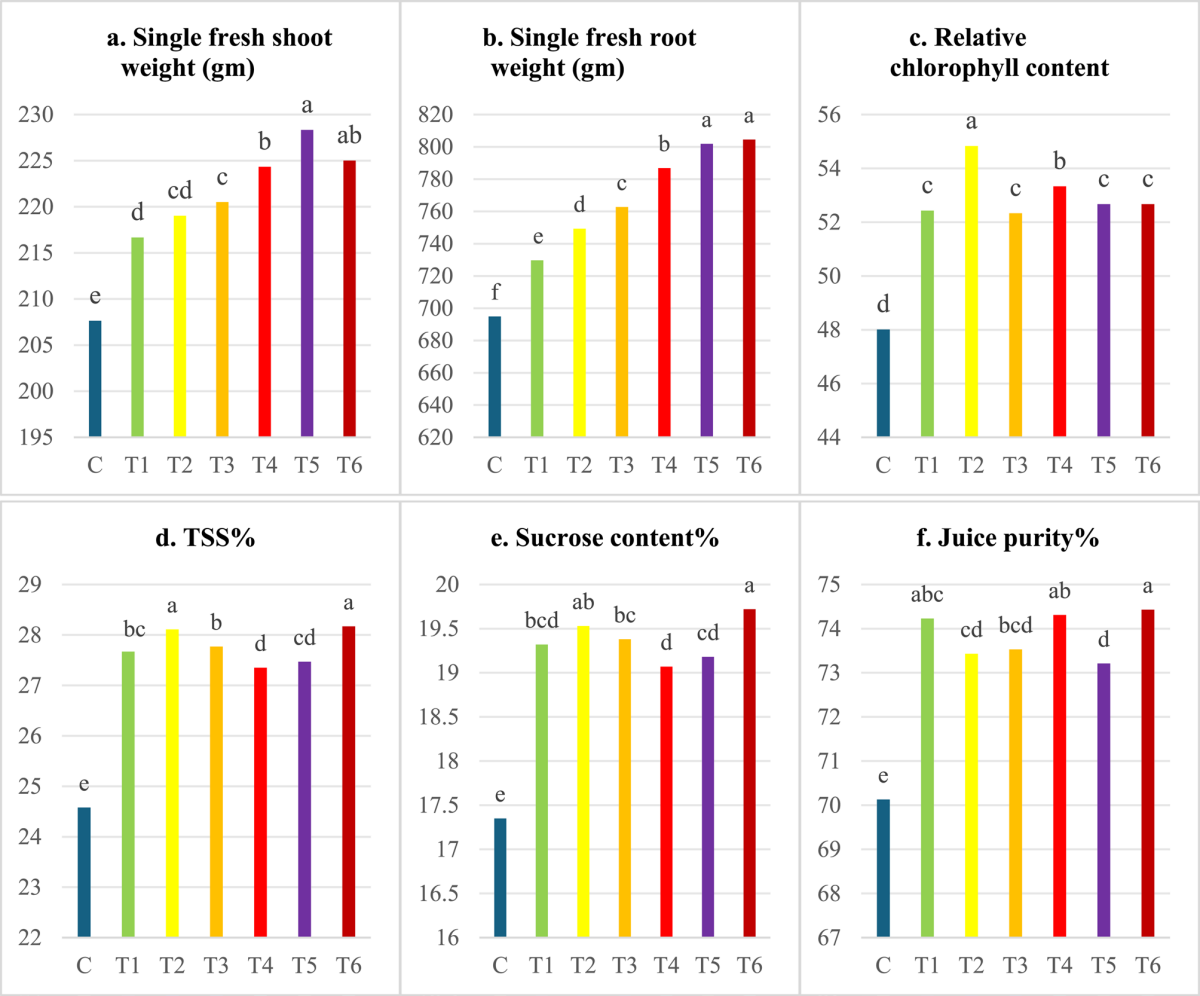
Effect of seed priming application of Ag, ZnONFs, and Ag/ZnONFs composite on single fresh weight of root and shoot, chlorophyll content, TSS, sucrose, and purity percentage in two sugar beet genotypes. The vertical bar represents the ± SE of means. Seed priming in water (C), in Ag NFs at 50 ppm (T1) and 100 ppm (T2), in ZnONFs at 50 ppm (T3) and 100 ppm (T4), and Ag/ZnONFs composite at 50 ppm (T5) and 100 ppm (T6)

Foliar application of Ag/ZnONFs composites (S5: 50 ppm and S6: 100 ppm) significantly enhanced juice quality parameters total soluble solids (TSS), sucrose content, and juice purity compared to other treatments. Specifically, at 50 ppm, increases were 17.36% (TSS), 20.67% (sucrose), and 2.69% (purity); at 100 ppm, 16.24%, 20.40%, and 4.47%, respectively, over the control. For seed priming (Fig. 6), all nanofertilizer treatments significantly improved plant traits compared to the control. The Ag/ZnONFs composite at 50 ppm (T5) and 100 ppm (T6) yielded the highest shoot (228.33 and 225 g) and root weights (801.83 and 804.51 g). T6 also recorded the highest TSS (28.17%), sucrose content (19.72%), and juice purity (74.43%) far exceeding control values (14.61%, 13.66%, and 6.13%, respectively).

These findings are consistent with previous studies reporting the effectiveness of nanofertilizers in improving sugar beet growth and quality traits. Dewdar, Abbas [12], and Elfattah Abd Elaleem [11] similarly observed enhanced development and yield upon nanofertilizer application. The observed increases in total soluble solids, sucrose content, and juice purity align with the findings of Hefny and Said [35], and Veronica, Guru [36], who reported that nanofertilizer applications significantly improve sugar-related quality traits in sugar beet. Furthermore, Liu and Lal [37] emphasized the role of nanofertilizers in enhancing nutrient absorption and reactivity, thereby supporting better plant growth and development.

The improved fresh weights of shoots and roots with AgNFs, particularly at 100 ppm, align with Latif, Ghareib [38], who reported that foliar application of AgNFs increased wheat growth parameters. The underlying mechanism may involve Ag NFs’ ability to inhibit ethylene signaling, as demonstrated in fenugreek by Rezvani, Sorooshzadeh [39], and their influence on plant morphology, which varies with nanoparticle size and shape. Syu, Hung [40] found that decahedral Ag NFs notably enhanced root growth in Arabidopsis. The increase in relative chlorophyll content with 50 ppm AgNFs agrees with Racuciu and Creanga [41], who observed similar trends in maize, where lower concentrations of AgNFs boosted chlorophyll, while higher doses led to inhibition.

The positive effect of ZnONFs on chlorophyll content is supported by previous studies [42, 43], which attributed this improvement to zinc’s role in enzymatic activity related to chlorophyll biosynthesis [44]. Zinc may also affect the availability of essential elements like iron and magnesium, further enhancing chlorophyll formation. Beyond chlorophyll enhancement, ZnONFs are known to stimulate the production of flavonoids, phenolics, and charantin, likely due to improved sugar metabolism [45], and upregulation of the shikimate-phenylpropanoid pathway [46]. ZnONFs may also influence calcium homeostasis and reactive oxygen species (ROS) signaling, contributing to a higher phytochemical profile, as reported in bitter gourd [47]. Mitogen-activated protein Kinase (MAPK) modulation is epigenetically regulated by nanoparticles, increasing the quantities of flavonoids and phenolics in metabolites. These cooperative processes lead to genetic transcriptome reprogramming.

The significant improvement in TSS, sucrose content, and juice purity with Ag/ZnONFs composite treatments is consistent with Masri and Hamza [48], who observed improved sugar beet productivity following foliar application of micronutrients. Likewise, Mekdad and Rady [49] demonstrated enhanced quality traits in sugar beet cultivars treated with iron, manganese, and zinc.

For seed priming treatments, the observed improvements are attributed to physiological responses during rehydration, such as increased antioxidant synthesis, activation of DNA repair systems [50], and protein production involved in cell division [51], which collectively promote early plant development and vigor. These effects support the findings on ZnONFs’ contribution to crop performance and quality enhancement [52].

The standard deviation was represented for each column. Means with the same letters are not significant according to Duncan’s test.

Figure 5. Effect of foliar application of Ag, ZnONF_S_ and Ag/ZnONFs composite on single fresh weight of root and shoot, Chlorophyll content, TSS %, sucrose %, and purity % in two sugars beets genotypes. The vertical bar represents ± SE of means. Water foliar spray (C), foliar spray of AgNF_S_ of 50 ppm (S1) and 100 ppm (S2), foliar spray ZnONF_S_ of 50 ppm (S3) and 100 ppm (S4), foliar spray (Ag/ZnONFs composite) of 50 ppm (S5) and 100 ppm (S6).

**Fig. 7:**
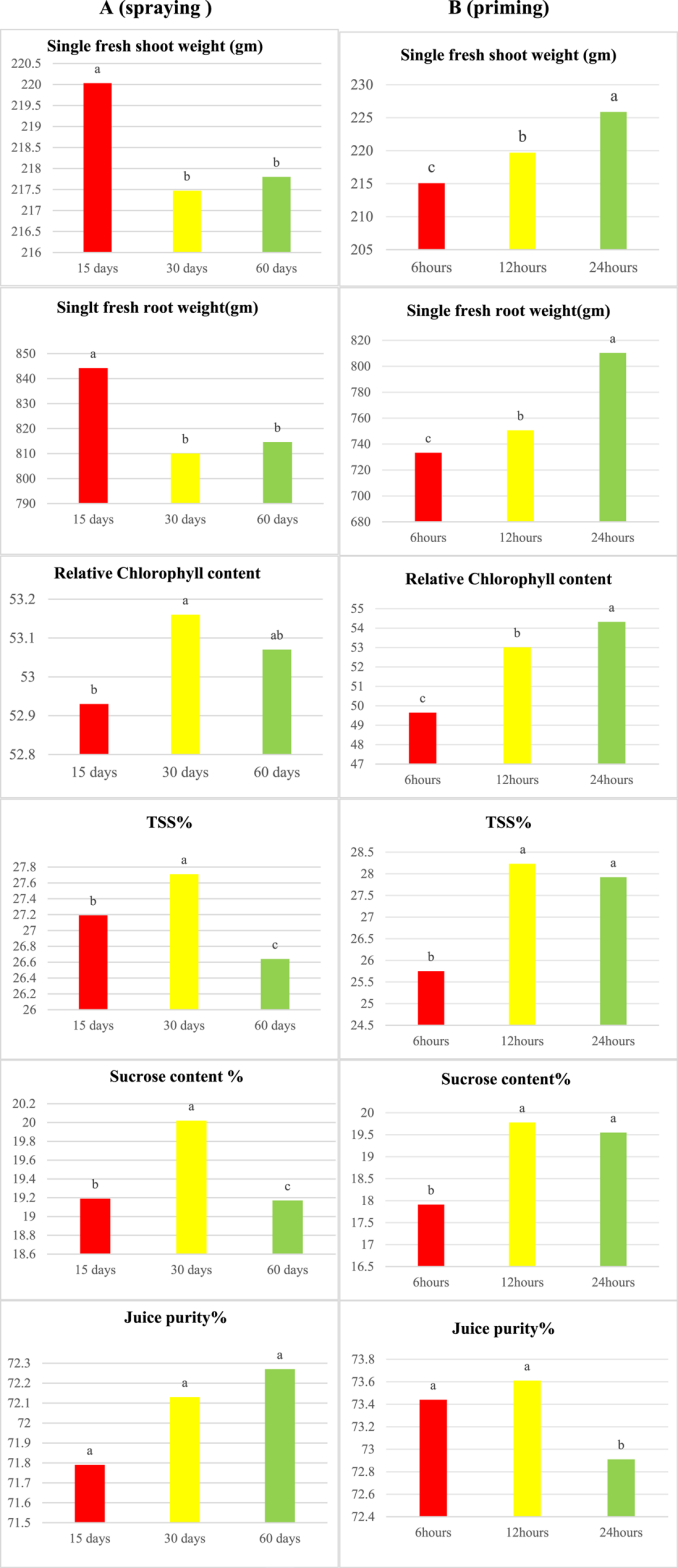
The value means of studied traits variety and dates (15,30 and 60 days) to foliar spraying and duration (6, 12, and 24 h) of seed priming with the Ag, ZnONFs, and Ag/ZnONFs composite treatments. The standard deviation was represented for each column. Means with the same letters are not significant according to Duncan’s test

The data presented in Fig. 7A indicate that foliar application of Ag, ZnONFs, and Ag/ZnONFs composite at 15 days after sowing resulted in a significant increase in the weight of single shoots and roots of sugar beet plants compared to the other two application dates. Conversely, foliar spraying of sugar beet plants with green nanoparticle treatments 30 days after sowing led to a significant increase in chlorophyll content, TSS, and sucrose percentages. However, there was an insignificant difference observed in purity percentage among the three dates of foliar application. Previous research in the field of zinc has shown that the application of this element at different stages of plant growth can have varied effects on plant performance [53]. Figure 7B presents our results, which indicate that seed priming with Ag, ZnONFs, and their composite for 24 h significantly improved all of the traits under investigation, except for purity%. However, there was an insignificant change in the percentages of sucrose and TSS after seed priming for either 12–24 h.

A (spraying) B (priming).

**Fig. 8:**
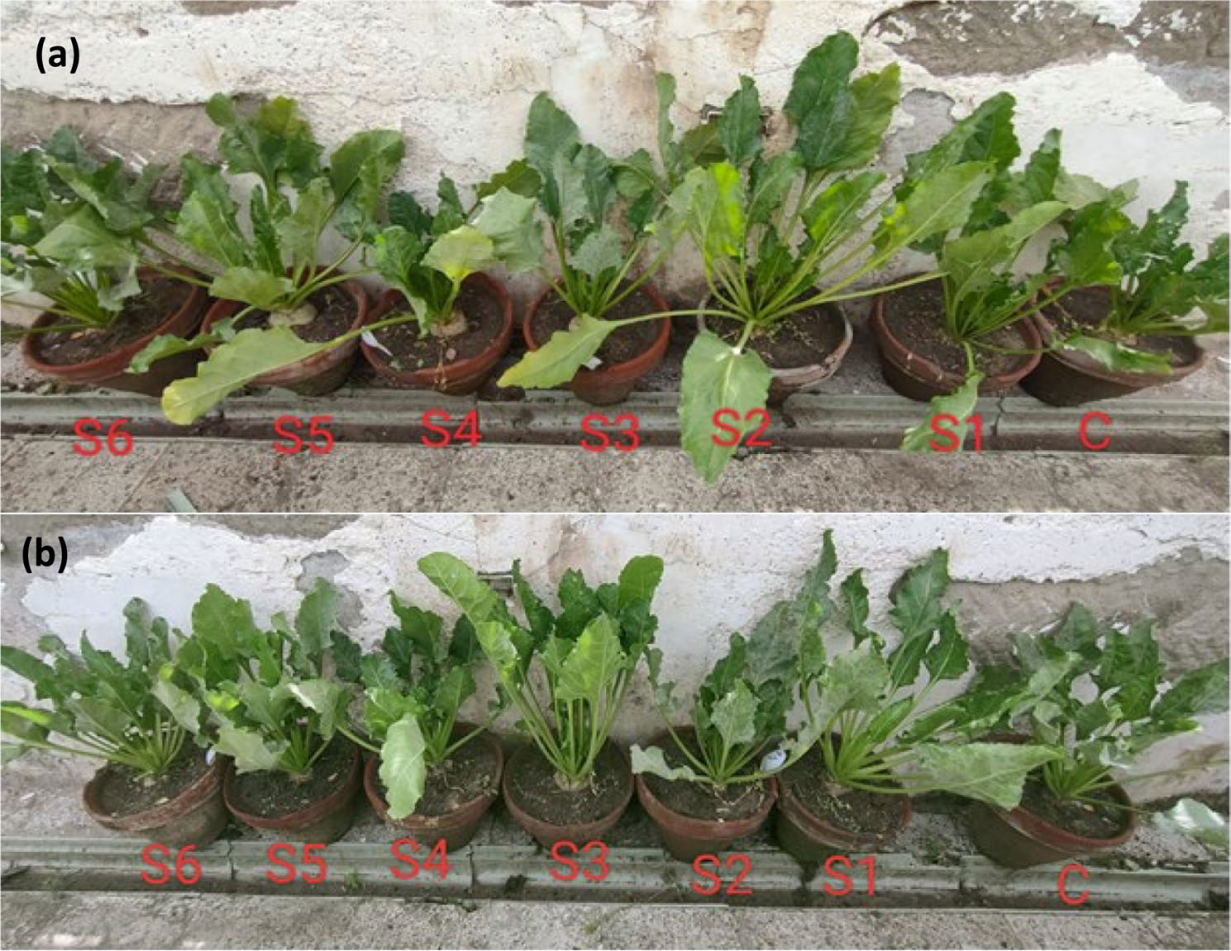
Effect of foliar application of Ag, ZnONFs, and composite on a single fresh shoot in Kawamaria sugar beet variety at 15 days (a) and 60 days (b). water foliar spray (C), foliar spray of AgNFs of 50 ppm (S1) and 100 ppm(S2), foliar spray ZnONFs of 50 ppm (S3) and 100 ppm (S4), foliar spray (Ag/ZnO composite) of 50 ppm (S5) and 100 ppm(S6)

Interaction effects revealed that foliar spraying with AgNFs at 100 ppm (S2) 15 days after sowing resulted in the highest single shoot and root weights. However, at sowing 30 and 60 days, the shoot and root weights decreased by 15 days for both Kawamira and Dema poly varieties (Table 5 and Fig. 8).

Regarding the interaction effects on TSS and sucrose percentages, the data in Table 5 indicated insignificant differences between foliar spraying with Ag/ZnONFs composite at 50 ppm (S5) at 15, 30, and 60 days after sowing. However, when the concentration was increased to 100 ppm, the percentage of TSS and sucrose significantly decreased at 15 and 60 days after sowing for the Dema poly variety. In contrast, TSS and sucrose percentages showed a higher and significant value when sprayed with Ag/ZnONFs composite at both 50 and 100 ppm at 30 days after sowing for the Kawamira variety. Cultivars exhibited significant differences in juice quality, root, and sugar yields in favour of the Toro and Farida cultivars compared with the cultivar [54].

The seed treatment with Ag/ZnONFs composite at 50 ppm resulted in the highest weight of single shoot and root, followed by the treatment with ZnONFs at 100 ppm for 24 h, for the Kawemira and Dema poly varieties. Similarly, the seeds primed with Ag/ZnONFs composite at 100 ppm for 12 h led to the highest percentages of TSS, sucrose, and juice purity for both studied varieties (Table 6).

Regarding the impact of interaction on the shoot weight of the Kawemira variety, the findings presented in Table 6 indicate that there was no significant difference in shoot weight when seeds were primed for 12–24 h using an Ag/ZnONFs composite at a concentration of 100 ppm. However, a noticeable increase in shoot weight was observed when the concentration was reduced to 50 ppm and the seeds were primed for 24 h.

The results in Table 6 showed that there was no significant difference in the sucrose content percentage between seeds primed with ZnONFs at 100 ppm for 12 and 24 h. However, when the concentration decreased to 50 ppm, seeds primed for 24 h produced a higher sucrose content percentage for the Kawemira variety.

**Table 5:** Effect of interaction between sugar beet varieties, nanofertilizer treatments, and the dates of foliar spraying on sugar beet growth and juice quality traits

Dates	T	Shoot fresh weight/gm		Root fresh weight/gm		Relative chlorophyll content		TSS%		Sucrose content%		Juice purity%	
		K*	D*	K	D	K	D	K	D	K	D	K	D
**15 days**	**C**	210.4	205.0	752.0	705.0	47.0	49.0	23.2	25.2	16.42	18.26	70.8	72.4
	**S1**	230.0	222.0	961.0	927.0	51.4	53.6	27.4	29.4	19.74	20.60	72.2	70.2
	**S2**	**240.0**	**231.0**	**995.6**	**984.4**	49.2	50.6	27.6	30.2	19.42	22.31	70.4	73.2
	**S3**	225.0	213.0	862.0	814.0	48.2	56.3	27.0	29.0	18.78	21.31	69.6	73.6
	**S4**	220.0	228.0	830.6	892.4	52.9	57.0	24.6	26.4	17.54	18.69	71.0	71.0
	**S5**	215.0	212.0	790.0	752.0	55.3	60.0	25.2	**30**.4	17.04	**22.92**	69.8	75.4
	**S6**	213.0	216.0	765.0	788.0	51.5	59.0	27.0	28.0	18.64	21.39	69.2	76.3
**30 days**	**S1**	218.2	222.4	826.0	854.0	48.3	58.5	25.4	27.0	17.79	19.48	70.0	72.2
	**S2**	230.4	220.4	924.4	845.0	57.6	53.2	27.2	29.2	18.88	21.32	69.6	73.2
	**S3**	220.0	216.4	807.0	778.0	48.6	60.4	26.4	28.6	18.83	20.18	71.2	72.4
	**S4**	223.4	225.2	920.4	872.4	49.1	60.2	28.2	28.6	20.00	20.96	70.8	73.2
	**S5**	216.0	213.4	775.6	758.0	49.6	57.9	**28.8**	**30.8**	**21.25**	**22.25**	73.8	72.4
	**S6**	213.0	210.2	766.2	756.0	49.9	55.0	**28.4**	31.0	**21.52**	22.91	75.8	73.8
**60 days**	**S1**	224.0	222.2	832.0	842.0	51.6	59.4	27.0	27.4	18.99	20.39	70.4	74.6
	**S2**	224.4	220.6	912.0	835.6	54.7	53.7	24.6	27.0	17.44	19.88	71.0	73.6
	**S3**	222.4	216.2	861.8	778.0	53.4	60.0	24.6	29.2	16.42	21.99	70.4	75.2
	**S4**	224.8	221.2	917.0	838.0	53.2	53.0	25.4	29.8	18.02	22.11	58.4	74.2
	**S5**	217.4	212.6	780.0	788.0	49.0	51.6	25.6	**29.6**	17.99	**21.88**	70.2	73.8
	**S6**	213.8	214.4	778.4	784.8	51.0	56.4	26.6	27.8	18.99	20.15	71.4	72.6
**LSD 0.05**		**4.330**	**1.635**	**21.435**	**1.966**	**0.441**	**0.981**	**1.085**	**1.270**	**1.085**	**1.270**	**ns**	**2.627**

Water foliar spray (C), foliar spray of AgNFS of 50 ppm (S1) and 100 ppm (S2), foliar spray ZnONPS of 50 ppm(S3) and 100 ppm (S4), foliar spray (Ag/ZnONFs composite) of 50 ppm (S5) and 100 ppm (S6). K* for the variety Kawamaria, D* for the variety for Dema Poly. T for treatment.

**Table 6:** Effect of interaction between sugar beet varieties, nanofertilizer treatments, and the dates of seed priming on sugar beet growth and juice quality traits

Dates	T	Shoot fresh weight/gm		Root fresh weight/gm		Relative chlorophyll content		TSS%		Sucrose content%		Juice purity%	
		K*	D*	K	D	K	D	K	D	K	D	K	D
**6 h**	**C**	212	203	755	635	48.2	47.3	23.9	25.0	16.80	17.62	69.4	71.0
	**T1**	216	208	763	645	52.6	49.0	25.0	26.0	17.50	17.90	73.8	73.0
	**T2**	217	210	780	660	50.0	50.0	26.3	26.3	17.96	18.00	73.2	74.0
	**T3**	216	213	766	680	49.0	48.0	25.2	26.4	17.70	18.06	72.6	72.4
	**T4**	220	216	790	695	50.0	49.0	25.6	26.5	17.87	18.12	74.4	76.8
	**T5**	219	219	772	690	51.0	50.0	25.4	26.4	17.90	18.10	72.2	74.0
	**T6**	221	221	795	700	50.0	51.0	26.0	27.0	18.20	18.90	74.8	76.6
**12 h**	**C**	212	202	759	618	48.3	47.7	24.4	24.8	17.16	17.50	69.4	70.4
	**T1**	214	218	765	650	52.0	52.0	27.0	28.0	18.90	19.60	72.6	74.0
	**T2**	216	220	782	682	57.0	55.0	29.0	30.0	20.30	21.00	74.6	75.8
	**T3**	217	222	790	715	53.0	51.0	27.0	28.0	18.90	19.60	72.4	72.8
	**T4**	222	226	809	732	54.0	52.0	28.0	29.0	19.60	20.30	75.0	75.2
	**T5**	226	225	820	762	56.0	54.0	29.0	30.0	20.30	21.00	73.0	75.2
	**T6**	228	227	840	784	55.0	55.0	**30.0**	**31.0**	**21.00**	**21.70**	**75.0**	**75.2**
**24 h**	**C**	213	203	760	642	48.5	48.0	24.6	25.3	17.20	17.81	70.0	69.8
	**T1**	220	224	785	770	55.0	54.0	30.0	30.0	21.00	21.00	76.2	75.8
	**T2**	223	228	797	795	59.0	58.0	28.0	29.0	19.60	20.30	70.2	73.8
	**T3**	225	230	820	805	57.0	56.0	30.0	30.0	21.00	21.00	76.6	74.4
	**T4**	230	232	870	825	58.0	57.0	27.0	28.0	18.90	19.60	72.0	72.4
	**T5**	**240**	**241**	**902**	**865**	52.0	53.0	27.0	27.0	18.90	18.90	72.2	72.6
	**T6**	228	225	878	830	53.0	52.0	28.0	27.0	19.60	18.90	73.2	71.8
**LSD 0.05**		**0.819**	**1.026**	**4.485**	**12.659**	**1.281**	**0.883**	**0.861**	**0.417**	**0.712**	**0.660**	**1.649**	**1.916**

Seed priming with water (C), Seed priming with Ag NFs of 50 ppm (T1) and 100 ppm (T2), foliar spray ZnONFs of 50 ppm (T3) and 100 ppm (T4), foliar spray (Ag/ZnONFs composite) of 50 ppm (T5) and 100 ppm (T6). K* for variety Kawamaria, D* for variety for Dema poly. T for treatment.

In Table 7, Pearson’s correlation analysis shows significant positive correlations among most of the studied parameters in sugar beet seeds priming with Ag, ZnONFs, and Ag/ZnONFs composite, except for the correlation between root weight and juice purity. Furthermore, Table 8 indicates positive and negative correlations among different parameters. The data in Table 8 demonstrates that the weight of the shoot and root of the plants after nanofertilizers foliar spring treatments were negatively correlated with juice purity.

**Table 7:** Pearson’s correlation coefficients among the investigated parameters of the studied sugar beet varieties, seeds priming treatment with ag, ZnONF_S_ and ag/znonfs composite

Parameters	Seeds priming					
	Shoot fresh weight/gm	Root fresh weight/gm	Relative chlorophyll content	TSS%	Sucrose content%	Juice purity%
	**1**	**2**	**3**	**4**	**5**	**6**
**1**	1	0.79^***^	0.64^***^	0.56^***^	0.54^***^	0.28^***^
**2**		1	0.56^***^	0.30^***^	0.32^***^	0.6^ns^
**3**			1	0.73^***^	0.68^***^	0.26^***^
**4**				1	0.91^***^	0.54^***^
**5**					1	0.49^***^
**6**						1

*Significant at 5% level (p ≤ 0.05)

**Significant at 1% level (p ≤ 0.01)

***Significant at 0.1% level (p ≤ 0.001), ns non-significant at 0.05 levels.

**Table 8:** Pearson’s correlation coefficients for studied traits in two sugar beet varieties treated with foliar application of ag, ZnONF_S_ and ag/znonfs composite

Parameters	Foliar spring					
	Shoot fresh weight/gm	Root fresh weight/gm	Relative chlorophyll content	TSS%	Sucrose content%	Juice purity%
	1	2	3	4	5	6
**1**	1	0.90^**^	0.17^**^	0.14^**^	0.05^ns^	−0.13^ns^
**2**		1	0.97^ns^	0.14^**^	0.04^ns^	−0.17^*^
**3**			1	0.43^***^	0.46^***^	0.13^ns^
**4**				1	0.94^***^	0.16^*^
**5**					1	0.33^***^
**6**						1

*Significant at 5% level (p ≤ 0.05)

**Significant at 1% level (p ≤ 0.01)

***Significant at 0.1% level (p ≤ 0.001), ns non-significant at 0.05 levels

## Conclusion

This study demonstrated the significant potential of green-synthesized silver (Ag), zinc oxide (ZnO), and Ag/ZnO nanofertilizers (NFs) in enhancing the growth and quality traits of *Beta vulgaris* L. (sugar beet). Both foliar spraying and seed priming methods effectively improved plant performance, with notable differences based on the application type and concentration. Foliar application of AgNFs at 100 ppm significantly increased shoot and root fresh weights, achieving 240 g and 995.6 g for Kawemira, and 231 g and 984 g for Dema Poly, respectively. In contrast, seed priming with Ag/ZnONFs at 50–100 ppm resulted in comparable or even superior results in terms of biomass accumulation and sugar quality, with TSS and sucrose percentages reaching up to 31% and 22.91%, respectively. Among the tested treatments, seed priming with Ag/ZnONFs composites at 100 ppm for 12–24 h emerged as the most effective and practical approach for improving sugar beet performance. This method required significantly lower nanofertilizer quantities compared to foliar spraying and offered ease of integration into existing farming systems.

## Data Availability

Data will be made available on request.

## References

[1] Aryal JP, et al. Climate change and agriculture in South Asia: adaptation options in smallholder production systems. Environ Dev Sustain. 2020;22(6):5045–75.

[2] Celik S. *The effects of climate change on human behaviors*, in *Environment, climate, plant and vegetation growth*. Springer; 2020. pp. 577–89.

[3] Pathak TB, et al. Climate change trends and impacts on California agriculture: a detailed review. Agronomy. 2018;8(3):25.

[4] Khan F, Pandey P, Upadhyay TK. Applications of nanotechnology-based agrochemicals in food security and sustainable agriculture: an overview. Agriculture. 2022;12(10):1672.

[5] Mgadi K, et al. Nanoparticle applications in agriculture: overview and response of plant-associated microorganisms. Front Microbiol. 2024;15:1354440. 38511012 10.3389/fmicb.2024.1354440 PMC10951078

[6] Beheiry HR, et al. Potassium spraying preharvest and nanocoating postharvest improve the quality and extend the storage period for acid lime (*Citrus aurantifolia* Swingle) fruits. Plants. 2023;12(22):3848. 38005744 10.3390/plants12223848 PMC10674589

[7] Abdelkhalek A, et al. *Ocimum basilicum*-mediated synthesis of silver nanoparticles induces innate immune responses against cucumber mosaic virus in squash. Plants. 2022;11(20):2707. 36297731 10.3390/plants11202707 PMC9609463

[8] Kiumarzi F, et al. Selenium nanoparticles (Se-NPs) alleviates salinity damages and improves phytochemical characteristics of pineapple mint (*Mentha suaveolens* Ehrh). Plants. 2022;11(10):1384. 35631809 10.3390/plants11101384 PMC9147120

[9] Khan MR, Rizvi TF. *Application of nanofertilizer and nanopesticides for improvements in crop production and protection*, in *Nanoscience and plant–soil systems*. Springer; 2017. pp. 405–27.

[10] Khan MR, Rizvi TF. *Application of nanofertilizer and nanopesticides for improvements in crop production and protection.* Nanoscience and plant–soil systems, 2017: pp. 405–427.

[11] Elfattah A, Elaleem H. Impact of nano-micronutrients as foliar fertilization on yield and quality of sugar beet roots. Pakistan J Biol Sciences: PJBS. 2020;23(11):1416–23. 10.3923/pjbs.2020.1416.1423 33274869

[12] Dewdar M, et al. Effect of nano micronutrients and nitrogen foliar applications on sugar beet (Beta vulgaris L.) of quantity and quality traits in marginal soils in Egypt. Int J Curr Microbiol Appl Sci. 2018;7(08):4490–8.

[13] Khan S, et al. The impact of silver nanoparticles on the growth of plants: the agriculture applications. Heliyon. 2023. 10.1016/j.heliyon.2023.e16928. 37346326 10.1016/j.heliyon.2023.e16928 PMC10279825

[14] Nejatzadeh F. Effect of silver nanoparticles on salt tolerance of *satureja hortensis* L. during *in vitro* and *in vivo* germination tests. Heliyon 7 , no. 2 (2021). 10.1016/j.heliyon.2021.e05981 33644433 10.1016/j.heliyon.2021.e05981 PMC7895726

[15] Zulfiqar F, et al. Nanofertilizer use for sustainable agriculture: advantages and limitations. Plant Sci. 2019;289:110270. 31623775 10.1016/j.plantsci.2019.110270

[16] Singh H, et al. Recent advances in the applications of nano-agrochemicals for sustainable agricultural development. Environ Sci Process Impacts. 2021;23(2):213–39. 33447834 10.1039/d0em00404a

[17] Singh RP, Handa R, Manchanda G. Nanoparticles in sustainable agriculture: an emerging opportunity. J Controlled Release. 2021;329:1234–48. 10.1016/j.jconrel.2020.10.051 33122001

[18] Singh A, et al. Zinc oxide nanoparticles: a review of their biological synthesis, antimicrobial activity, uptake, translocation and biotransformation in plants. J Mater Sci. 2018;53(1):185–201.

[19] Mao-Hong L, et al. Effects of zinc oxide nanoparticles on germination and seedling growth of two vegetables. J Agric Resour Environ. 2021;38(1):72.

[20] Lala S. Nanoparticles as elicitors and harvesters of economically important secondary metabolites in higher plants: a review. IET Nanobiotechnol. 2021;15(1):28–57. 34694730 10.1049/nbt2.12005 PMC8675826

[21] Shokohian A, Omidi H. Sugar beet (Beta vulgaris L.) germination indices and physiological properties affected by priming and genotype under salinity stress. Volume 1. Notulae Botanicae Horti Agrobotanici Cluj-Napoca; 2021. 1.

[22] Hassnein A, et al. Effect of nano fertilization on sugar beet. Al-Azhar J Agric Res. 2019;44(2):194–201.

[23] Garibo D, et al. Green synthesis of silver nanoparticles using *Lysiloma acapulcensis* exhibit high-antimicrobial activity. Sci Rep. 2020;10(1):12805. 32732959 10.1038/s41598-020-69606-7 PMC7393152

[24] Naiel B, et al. Green synthesis of zinc oxide nanoparticles using sea lavender (*Limonium pruinosum* L. Chaz.) extract: characterization, evaluation of anti-skin cancer, antimicrobial and antioxidant potentials. Sci Rep. 2022;12(1):20370. 36437355 10.1038/s41598-022-24805-2 PMC9701696

[25] Cooke DA, Scott J. The sugar beet crop. Springer Science & Business Media; 2012.

[26] Carruthers A, Oldfield J. Methods for the assessment of beet quality. Int Sugar J. 1961;63:72–4.

[27] Carruthers A, Oldfield J, Teague H. The removal of interfering ions in the determination of betaine in sugar-beet juices and plant material. Analyst. 1960;85(1009):272–5.

[28] Snedecor GW, Cochran WG. Statistical methods. 1989. Ames, IA, USA: Iowa State College; 1980.

[29] Waller RA, Duncan DB. A bayes rule for the symmetric multiple comparisons problem. J Am Stat Assoc. 1969;64(328):1484–503.

[30] Bello BA, et al. Anticancer, antibacterial and pollutant degradation potential of silver nanoparticles from *Hyphaene thebaica*. Biochem Biophys Res Commun. 2017;490(3):889–94. 28648600 10.1016/j.bbrc.2017.06.136

[31] Karam ST, Abdulrahman AF. Green synthesis and characterization of ZnO nanoparticles by using thyme plant leaf extract. Photonics. MDPI; 2022.

[32] Alla N, Mohamed A, Zalat S. Effect of soil and foliar application of nitrogen fertilization on sugar beet. J Plant Prod. 2002;27(3):1343–51.

[33] Erciyes H, Bulut S, Arslan M. Yield and quality characteristics of sugar beet cultivars under continental Climatic conditions. Curr Trends Nat Sci 5, no. 9. 2016.

[34] Abd El-Lateef HM, Shalabi K, Tantawy AH. Corrosion inhibition and adsorption features of novel bioactive cationic surfactants bearing benzenesulphonamide on C1018-steel under sweet conditions: combined modeling and experimental approaches. J Mol Liq. 2020;320:114564.

[35] Hefny YA, Said AA. Effect of nano-micronutrients fertilization on yield and quality of some sugar beet varieties under early and late sowing dates. Egypt J Agron. 2021;43(1):55–68.

[36] Veronica N, et al. Role of nano fertilizers in agricultural farming. Int J Environ Sci Technol. 2015;1(1):1–3.

[37] Liu R, Lal R. Potentials of engineered nanoparticles as fertilizers for increasing agronomic productions. Sci Total Environ. 2015;514:131–9. 25659311 10.1016/j.scitotenv.2015.01.104

[38] Latif H, Ghareib M, Tahon M. Phytosynthesis of silver nanoparticles using leaf extracts from *Ocimum basilicum* and *Mangifira indica* and their effect on some biochemical attributes of *Triticum aestivum*. Gesunde Pflanz. 2017. 10.1007/s10343-017-0385-9.

[39] Rezvani N, Sorooshzadeh A, Farhadi N. Effect of nano-silver on growth of saffron in flooding stress. Int J Agric Biosyst Eng. 2012;6(1):11–6.

[40] Syu Y-y, et al. Impacts of size and shape of silver nanoparticles on Arabidopsis plant growth and gene expression. Plant Physiol Biochem. 2014;83:57–64. 25090087 10.1016/j.plaphy.2014.07.010

[41] Racuciu M, Creanga D. TMA-OH coated magnetic nanoparticles internalized in vegetal tissue. Rom J Phys. 2007;52(3/4):395.

[42] Khodakovskaya MV et al. *Complex genetic, photothermal, and photoacoustic analysis of nanoparticle-plant interactions.* Proceedings of the National Academy of Sciences, 2011. 108(3): pp. 1028–1033. 10.1073/pnas.1008856108 PMC3024702 21189303

[43] Tarafdar J, et al. Development of zinc nanofertilizer to enhance crop production in Pearl millet (*Pennisetum americanum*). Agric Res. 2014;3:257–62.

[44] Lebedev N, Timko MP. Protochlorophyllide photoreduction. Photosynth Res. 1998;58(1):5–23.

[45] Sun L, et al. Physiological, transcriptomic, and metabolomic analyses reveal zinc oxide nanoparticles modulate plant growth in tomato. Environ Sci Nano. 2020;7(11):3587–604.

[46] Zhao L, et al. Nano-biotechnology in agriculture: use of nanomaterials to promote plant growth and stress tolerance. J Agric Food Chem. 2020;68(7):1935–47. 32003987 10.1021/acs.jafc.9b06615

[47] Kruszka D, et al. Untargeted metabolomics analysis reveals the elicitation of important secondary metabolites upon treatment with various metal and metal oxide nanoparticles in *Hypericum perforatum* L. cell suspension cultures. Ind Crops Prod. 2022;178:114561.

[48] Masri M, Hamza M. Influence of foliar application with micronutrients on productivity of three sugar beet cultivars under drip irrigation in sandy soils. World J Agric Sci. 2015;11(2):55–61.

[49] Mekdad A, Rady M. *Response of Beta vulgaris L. to nitrogen and micronutrients in dry environment.* 2016.

[50] Farooq M, et al. Seed priming in field crops: potential benefits, adoption and challenges. Crop Pasture Sci. 2019;70(9):731–71.

[51] Shelar A, et al. Sustainable agriculture through multidisciplinary seed nanopriming: prospects of opportunities and challenges. Cells. 2021;10(9):2428. 34572078 10.3390/cells10092428 PMC8472472

[52] Waqas Mazhar M, et al. Seed priming with iron oxide nanoparticles raises biomass production and agronomic profile of water-stressed flax plants. Agronomy. 2022;12(5):982.

[53] Alloway BJ. *Micronutrients and crop production: an introduction*, in *Micronutrient deficiencies in global crop production*. Springer; 2008. pp. 1–39.

[54] Azzazy HM, Mansour MM, Kazmierczak SC. From diagnostics to therapy: prospects of quantum dots. Clin Biochem. 2007;40(13–14):917–27. 17689518 10.1016/j.clinbiochem.2007.05.018

